# Alleviation of DSS-induced colitis via bovine colostrum-derived extracellular vesicles with microRNA *let-7a-5p* is mediated by regulating *Akkermansia* and **β**-hydroxybutyrate in gut environments

**DOI:** 10.1128/spectrum.00121-23

**Published:** 2023-11-15

**Authors:** Daye Mun, Minkyoung Kang, Minhye Shin, Hye Jin Choi, An Na Kang, Sangdon Ryu, Tatsuya Unno, Brighton E. Maburutse, Sangnam Oh, Younghoon Kim

**Affiliations:** 1 Department of Agricultural Biotechnology and Research Institute of Agriculture and Life Science, Seoul National University, Seoul, South Korea; 2 Department of Functional Food and Biotechnology, Jeonju University, Jeonju, South Korea; 3 Department of Microbiology, College of Medicine, Inha University, Incheon, South Korea; 4 Division of Evironmental Meterials, Honam National Institute of Biological Resources, Mokpo, South Korea; 5 Department of Microbiology, Chungbuk National University, Cheongju, South Korea; 6 Department of Animal Production Sciences, Marondera University of Agricultural Sciences & Technology, Marondera, Zimbabwe; National Health Research Institutes, Zhunan, Taiwan

**Keywords:** bovine colostrum, extracellular vesicles, microbiome, metabolome, bta-*let-7a-5p*

## Abstract

Inflammatory bowel disease (IBD) is an inflammatory disease caused by the interaction of environmental and genetic factors, including diet and microbiota. Recent studies have suggested the effects of extracellular vesicles (EVs) on controlling host intestinal immune responses, although the specified mechanisms have not yet been fully elucidated. In the current study, we investigated the effects of EVs isolated from bovine colostrum on attenuating dextran sodium sulfate-induced colitis in mice. Multiomic approaches were used to assess host gene expression and gut microbiota profiles and to explain the roles of EVs in modulating the host immune system. Transcriptomic analyses suggested a possible immunoregulatory mechanism mediated by the cell cycle regulator CDK6 and pro-inflammatory responses. The gut microbiome composition and fecal metabolite profiles showed that EV supplementation promoted the microbial abundance of *Akkermansia* and short-chain fatty acid production in mice with colitis. Further *in vivo* analysis revealed that β-hydroxybutyrate and *Akkermansia muciniphila*, which are elevated by the administration with EVs, are involved in lessening colitis through modulating the immune system. Additionally, the bta-let-7a-5p, one of the most abundant microRNAs in bovine colostrum-derived EVs, alleviated the indications of colitis in mice by modulating their intestinal immunity and by regulating the cell cycle-related target gene Cdk6. Overall, these results suggest that bovine colostrum EVs and bta-let-7a-5p could be promising alternative strategies for preventing the onset of IBD.

**IMPORTANCE:**

Even though studying on the possible involvement of extracellular vesicles (EVs) in host-microbe interactions, how these relationships mediate host physiology has not clarified yet. Our current findings provide insights into the encouraging benefits of dietary source-derived EVs and microRNAs (miRNAs) on organic acid production and ultimately stimulating gut microbiome for human health, suggesting that supplementation of dietary colostrum EVs and miRNAs is a novel preventive strategy for the treatment of inflammatory bowel disease.

## INTRODUCTION

Inflammatory bowel disease (IBD) is a disorder characterized by chronic inflammation of the gastrointestinal (GI) tract, including Crohn’s disease and ulcerative colitis. It is a long-lasting and recurrent immune-mediated disease that typically begins in young adulthood and has a lifelong course (1, 2). The prevalence of IBD has increased markedly in many countries, imposing a substantial social and economic burden on governments as well as individuals’ quality of life (3). The cause of IBD remains unclear, but recent studies have suggested an inappropriate immune response to environmental factors that alter the gut microbiota in a genetically susceptible host (4).

Extracellular vesicles (EVs) are particles with a lipid bilayer naturally released from a cell that carry a cargo of proteins, lipids, metabolites, and nucleic acids (5, 6). Based on accumulating evidence, EVs from milk play a critical role in intestinal maturation and function in the host (7). Administration of milk EVs supports intestinal cell growth, attenuates apoptosis, and increases goblet cell numbers and mucin production (7, 8). These studies have proposed mechanisms by which milk EVs regulate intestinal immune responses via immunomodulatory signal transduction, including the TLR4/NF-κB, p53, MAPK, and mTORC1 pathways (7, 9, 10). However, despite their apparent importance, little is known about the specific mechanisms and possible roles of these EVs in host intestinal immune responses.

The gut microbiota is a crucial environmental factor contributing to host physiology, especially intestinal fitness and functions (11). Alterations in the community composition modify their metabolic output, resulting in the subsequent initiation of human diseases, including IBD (4). EVs contain diverse cargo that are intricately involved in cell-cell signaling and cross-kingdom communication with the gut microbiome (12). Studies have identified that the absence of EVs decreases the diversity of the recipient’s intestinal microbiome and their supplementation alters bacterial communities (13, 14). Although these observations indicate the possible involvement of EVs in host-microbe interactions, how microbial changes mediate host physiology is not clarified yet.

EVs consist of various types of biological products, including proteins, lipids, and microRNAs (miRNAs). miRNAs are small nucleic acids ranging from 18 to 22 nucleotides that play a role in regulating posttranscriptional gene regulation and transferring signals into the host (15). Previous studies have reported that miRNA has been associated with a variety of physiological functions, including the regulation of the immune system (16). bta-let-7a-5p is one of the most abundant miRNAs in both human and bovine colostrum EVs (17). According to previous studies, let-7a-5p was significantly lower expressed in colorectal cancer cells, and reduced let-7a-5p was reported to inhibit cancer cell apoptosis and was suggested as a biomarker for colorectal cancer, suggesting that let-7a-5p may be associated with the regulation of inflammation and colitis (18, 19). However, studies on whether bovine colostrum-derived bta-let-7a-5p has a protective effect on DSS-derived colitis and the understanding of its mechanism are still insufficient.

In this study, we investigated the properties and function of bovine colostrum-derived EVs and bta-let-7a-5p in mouse models of dextran sodium sulfate (DSS)-induced colitis. Based on the results obtained using multiomics approaches, including metagenomics, metabolomics, and transcriptomics, we propose a probable protective mechanism of the effect of bovine colostrum EVs on DSS-induced intestinal immune disorder, which is strongly associated with alterations in the gut microbiota and miRNA-associated gene regulation.

## RESULTS

### Bovine colostrum-derived EVs improve the symptoms of DSS-induced colitis

**Fig 1:**
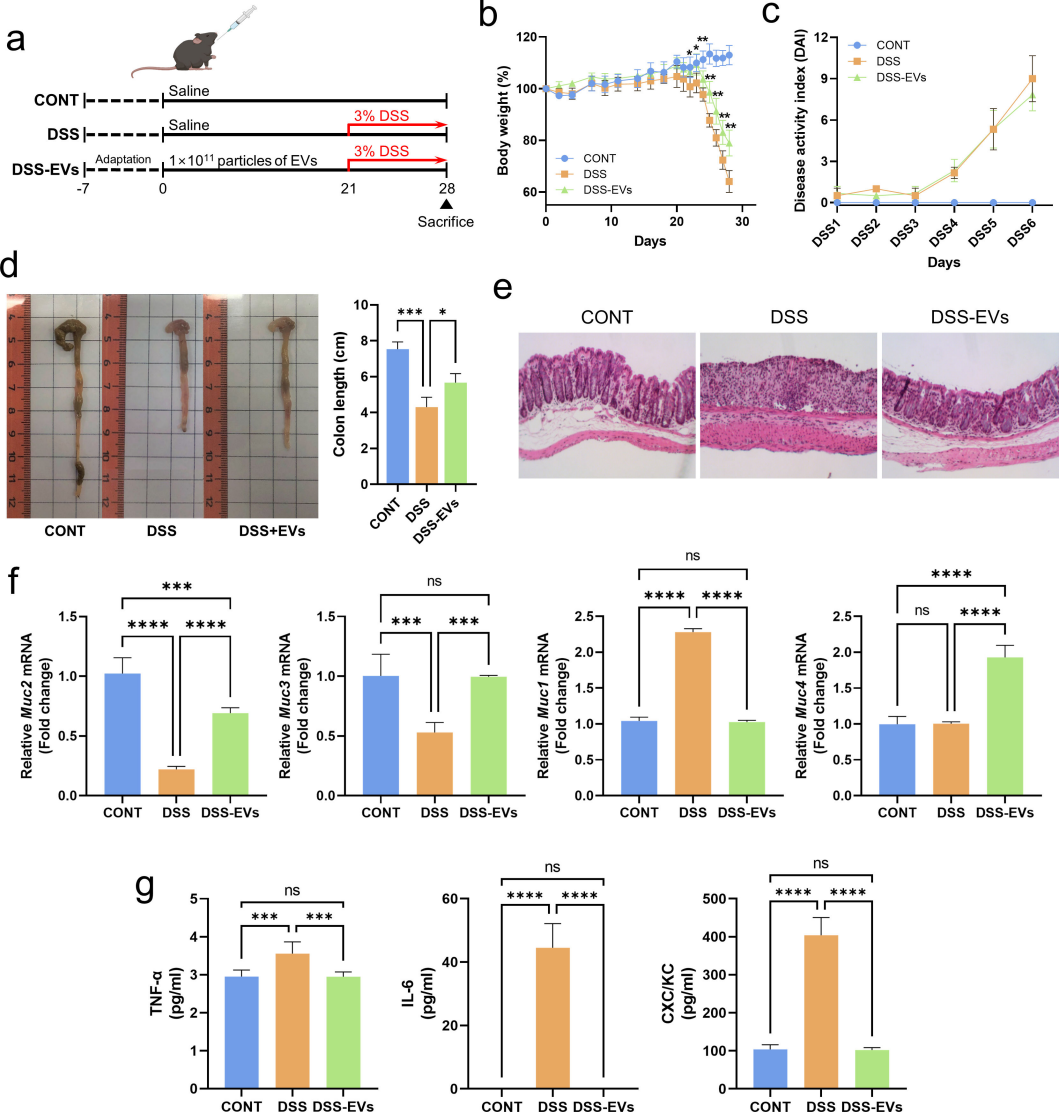
Bovine colostrum-derived EVs improve the gastrointestinal health of mice with DSS-induced colitis. (**a**) Bovine colostrum-derived EVs (1 × 10^11^ particles/mouse) were administered by oral gavage daily for 3 weeks and subsequent colitis induction at the last week by 3% DSS concurrent with EV administration. (**b**) Body weight changes in mice during EV treatment for 3 weeks and DSS induction for 1 week. (**c**) Disease activity index. (**d**) Representative colons (left panel) and colon length (right panel). (**e**) Representative histological H&E staining of sections of the colon. (**f**) The expression of genes (*Muc2*, *Muc3*, *Muc1*, and *Muc4*) associated with mucin production was analyzed using quantitative real-time PCR (qRT-PCR). (**g**) Serum levels of the pro-inflammatory cytokines TNF-α, IL-6, and CXC/KC. Data represent averages from 10 replicates, and error bars represent standard deviations. Statistical analyses comparing differences between the two groups were performed using Student’s *t*-test, and differences among the groups were performed using one-way analysis of variance (ANOVA) and Tukey’s multiple comparison test. Asterisks represent statistical significance at *P* < 0.05 (*), *P* < 0.01 (**), *P* < 0.001 (***), and *P* < 0.0001 (****). CONT, saline control; DSS, DSS-treated control; DSS-EVs, DSS induction with EV treatment; CONT-EVs, only EV treatment without DSS treatment.

Recent evidence has indicated that EVs play a key role in the intestinal function of humans and murine models (20). However, their systemic mechanism of action is not clearly understood. This study first aimed to assess the effects of bovine colostrum EVs on the symptoms of DSS-induced colitis murine model. Considering the preventive effects of EVs on ulcerative colitis-like pathologies, mice were treated with saline or EVs (1 × 10^11^ particles/mouse) were subjected to oral gavage for 3 weeks, and colitis was induced in mice using 3% DSS for 6 days (Fig. 1a). Both groups exposed to DSS showed a marked decrease in body weight compared with the control group (Fig. 1b). Although the supplementation of EVs in non-DSS mice did not affect the body weight change, the EV treatment attenuated weight loss in mice with DSS-induced colitis. In addition, mice in the DSS-EV group were more active and had less diarrhea than those in the DSS control group (Video S1). These results indicate a potential role for EVs in protecting against weight loss caused by DSS treatment.

We next measured the disease activity index (DAI) score and the colon length as a biological marker of colonic inflammation. Although there was no significant difference in the DAI scores of the DSS group and the DSS-EVs group (Fig. 1c), compared with the average colon length of the DSS-induced control group, the DSS group treated with EVs showed a significantly longer colon length (Fig. 1d). As shown in Fig. 1e, histological staining of hematoxylin-and-eosin (H&E)-stained tissue indicates an inflammatory effect of DSS on the colon tissue, showing a marked disintegration of the epithelium, substantial loss of crypts, and a high rate of mononuclear cell and granulocyte infiltration into the mucosa and submucosa. However, treatment with EVs clearly decreased the severity of these indicators of injury to the colon, suggesting that bovine colostrum EVs have a protective effect against colitis.

The intestinal epithelium is covered with a mucosal layer that acts as a physical barrier, and inflammatory bowel disease is characterized by disruption of this mucus layer. To determine whether administration of EVs to a DSS-induced colitis model affects the integrity of the intestinal mucosal layer, the mRNA expression level of mucin-producing genes was investigated. DSS treatment downregulated the expression of *Muc2* and *Muc3*, but the administration of EVs restored their expression (Fig. 1f). In contrast to *Muc2* and *Muc3*, higher *Muc1* expression was detected in mice with DSS-induced colitis but was reduced by the EV treatment. *Muc4* expression was upregulated only in the DSS-EV mouse group. The notable maintenance of *Muc2* expression in this study when DSS is administered together with EVs was an interesting finding given the involvement of Mucin 2 in the formation of a gel-like protective layer along the gut. Decreased expression of *Muc1*, which is commonly associated with increased disease states when it is expressed at high levels, also suggested health benefits in mice receiving DSS with EVs (21). These observations suggest that oral administration of bovine colostrum EVs attenuates DSS-induced colitis in mice.

### Bovine colostrum EVs reduce pro-inflammatory cytokines in DSS-colitis mice

Next, we measured the expression levels of three selected cytokines, TNF-α, interleukin-6 (IL-6), and CXCL1/KC, in mouse serum to assess the possible anti-inflammatory effects of EVs on the DSS-induced colitis model. Consistent with the body weight and colon length retrieved, serum levels of the cytokines TNF-α and IL-6 increased in the DSS-treated control mice compared with those of the group that received DSS with EVs (Fig. 1g). The expression levels of these cytokines in the DSS-EVs group were similar to those in control mice treated with phosphate-buffered saline (PBS) alone without DSS. The expression of IL-6, the major cytokine produced in the colitis (22), was below the detectable level in the control and DSS-EV mice, while it was abundant in the DSS-treated mice. The serum level of CXCL1 increased more than twofold in the mice treated with DSS alone compared with the non-DSS-induced control mice, and treatment with EVs reduced its expression to the normal level. In addition, when EVs were orally administered to mice for 4 weeks that did not induce colitis, there was no significant difference in the overall intestinal health and immune markers with the normal control group (not shown). Altogether, we confirmed that bovine colostrum EVs attenuated pro-inflammatory responses in mice with DSS-induced colitis, contributing to the restoration of colon fitness.

### The transcriptomic analysis reveals the immunomodulatory roles of EVs in regulating the cell cycle in mice with DSS-induced colitis

**Fig 2:**
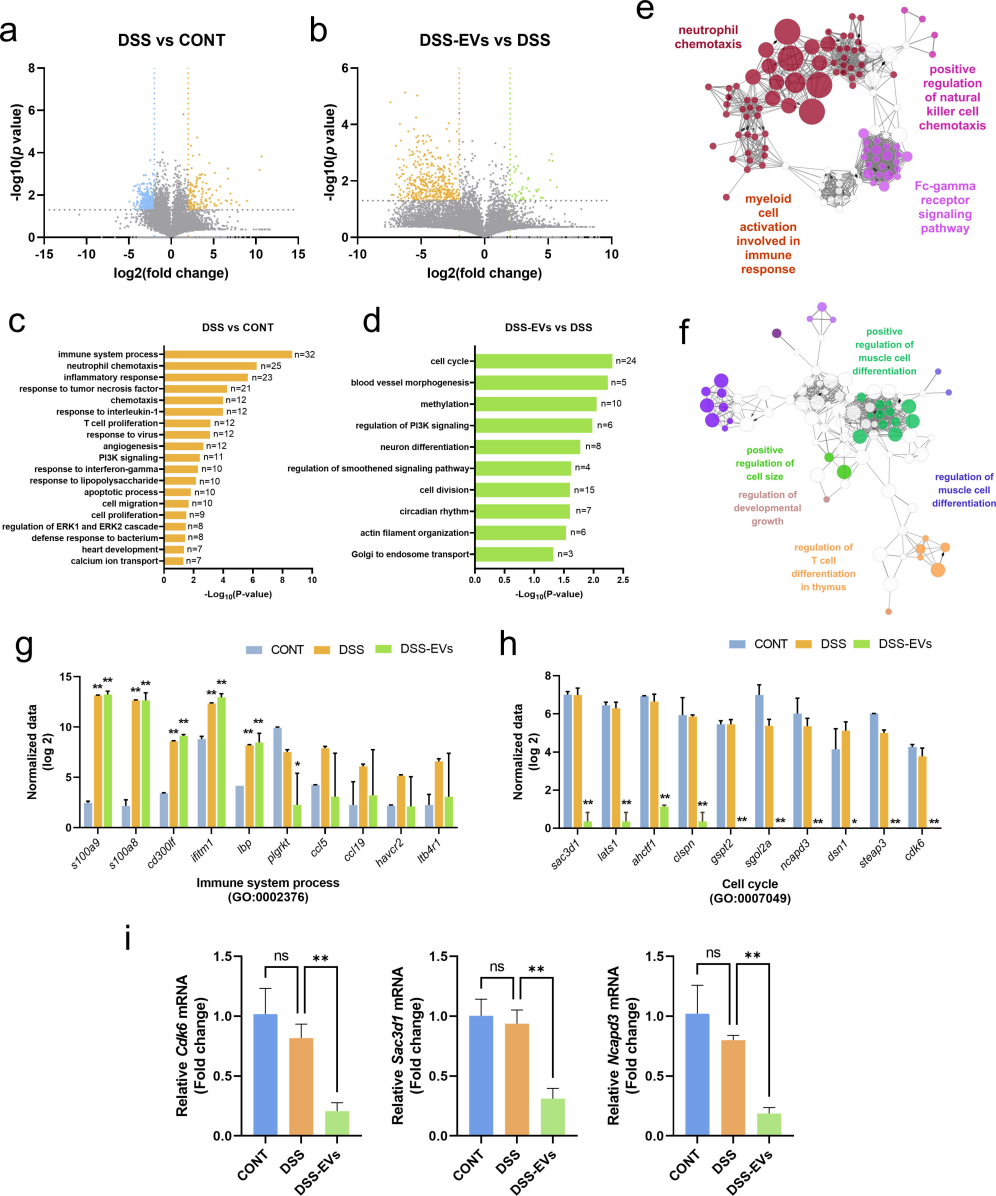
The transcriptomic analysis reveals the immunomodulatory roles of EVs in regulating the cell cycle in mice with DSS-induced colitis. (**a and b**) Volcano plots of differentially expressed genes in mouse intestines between CONT and DSS (**a**) or between DSS and DSS-EV groups (**b**). Blue and green dots indicate upregulated genes in the CONT group compared with the DSS group and in the DSS-EV group compared with the DSS group, respectively. Orange dots indicate upregulated genes in the DSS group compared with the CONT (**a**) or DSS-EVs group (**b**). Colored dots were selected with cutoff criteria of *P* < 0.05 and absolute [log 2 (fold change)] > 1. (**c and d**) DAVID-based Gene Ontology (GO) analysis of RNA-seq results between CONT and DSS groups (**c**) or between DSS and DSS-EV groups (**d**). Enriched terms in GO biological functions were annotated. (**e and f**) Enriched GO network group visualization using the ClueGo plugin of Cytoscape. Functionally grouped networks were generated based on GO immune system processes between CONT and DSS groups (**e**) and based on GO biological functions between DSS- and DSS-EV groups (**f**). Only the most significant term in each group is presented using ClueGo. (**g and h**) Top 10 most differentially expressed genes between CONT- and DSS-EV groups (**g**) and between DSS- and DSS-EV groups (**h**). (**i**) The mRNA expression (*Cdk6*, *Sac3d1*, and *Ncapd3*) associated with cell cycle was analyzed using qRT-PCR. Data represent averages, and error bars represent standard deviations. Statistical analyses among the groups were performed using one-way ANOVA and Dunnett’s multiple comparison test for comparing with the CONT group and Tukey’s multiple comparison test for mRNA expression. Asterisks represent statistical significance at *P* < 0.05 (*) and *P* < 0.01 (**). CONT, saline control; DSS, DSS-treated control; DSS-EVs, DSS induction with EV treatment.

We performed an RNA-sequencing (RNA-seq) analysis of the large intestine to investigate the effect of EV supplementation on global transcriptional changes that are possibly associated with changes in the gut microbiota composition. The differentially expressed genes (DEGs) were identified to show significant, greater than twofold changes in transcript expression (*P* < 0.05, Fig. 2a and b). One hundred forty-two and 411 genes were up- and downregulated, respectively, in the DSS group compared with the saline control group. EV supplementation resulted in 449 DEGs, with 40 and 409 upregulated and downregulated genes identified in the DSS-induced mouse groups, respectively. The most significant DE genes induced by DSS included *S100a9*, *Ifitm1*, and *Lbp*, while EV treatment of DSS-induced mice resulted in the differential expression of *Sac3d1*, *Ncapd3*, and *Cdk6* (Table S2).

A gene enrichment analysis was performed using DAVID, including Gene Ontology term and pathway enrichment analyses, to obtain further insights into the functions of the identified DEGs. Regarding GO biological process, the genes that were differentially expressed in the DSS samples compared with the control group were mainly enriched in the immune system process, inflammatory response, apoptotic process, and cell proliferation (Fig. 2c). Most of the enriched genes were upregulated in the DSS group, except for genes related to heart development and calcium ion transport. Moreover, EV administration to DSS-induced mice downregulated the expression of genes involved in the cell cycle, cell division, methylation, and neuronal differentiation (Fig. 2d). Functional implications of the DEGs were further analyzed based on the GO immune system and biological processes and visualized using ClueGO in the Cytoscape platform. DSS-induced genes were enriched in multiple functions related to chemotaxis and positive regulation of innate immune response (upregulated DE genes in DSS compared with CONT; Fig. 2e). Compared with DSS-EV and DSS samples, treatment with EVs resulted in the downregulation of genes related to cell differentiation and developmental growth (Fig. 2f).

In the DSS samples, the expression of genes involved in inflammatory processes and innate immune responses, including *S100a9*, *S100a8*, and *Havcr2*, was more highly upregulated than that in CONT mouse intestines (Fig. 2g). Interestingly, the increased expression of several genes (*Ccl5*, *Havcr2*, and *Ltb4r1*) in the DSS-induced samples was restored to the normal range by EV treatment. These genes function as chemokines and chemokine receptors and are upregulated in T cells from old mice compared with young mice, suggesting that EV treatment rescues the age-associated inflammation induced by DSS (23). Meanwhile, genes involved in the cell cycle, differentiation, and mitotic centrosome architecture, including *Sac3d1*, *Ahctf1*, and *Cdk6*, were significantly downregulated in the DSS-EV group compared with the DSS group without EV treatment (Fig. 2h). Validation of the sequencing-based analysis result was carried out as a quantitative analysis, and the expression of genes related to the cell cycle was decreased when EVs were treated. (Fig. 2i). Notably, these changes in gene expression were not observed in the mice treated with EVs alone, indicating that the effects of the EV treatment on cell cycle regulation depended on the inflammatory status (Fig. S1). Taken together, the EV treatment downregulated genes associated with cell cycle regulation in mice with DSS-induced colitis, which would be associated with immunomodulatory activities.

### The fecal microbiota analysis revealed a high abundance of *Akkermansia* in EV-administered DSS-induced mice

**Fig 3:**
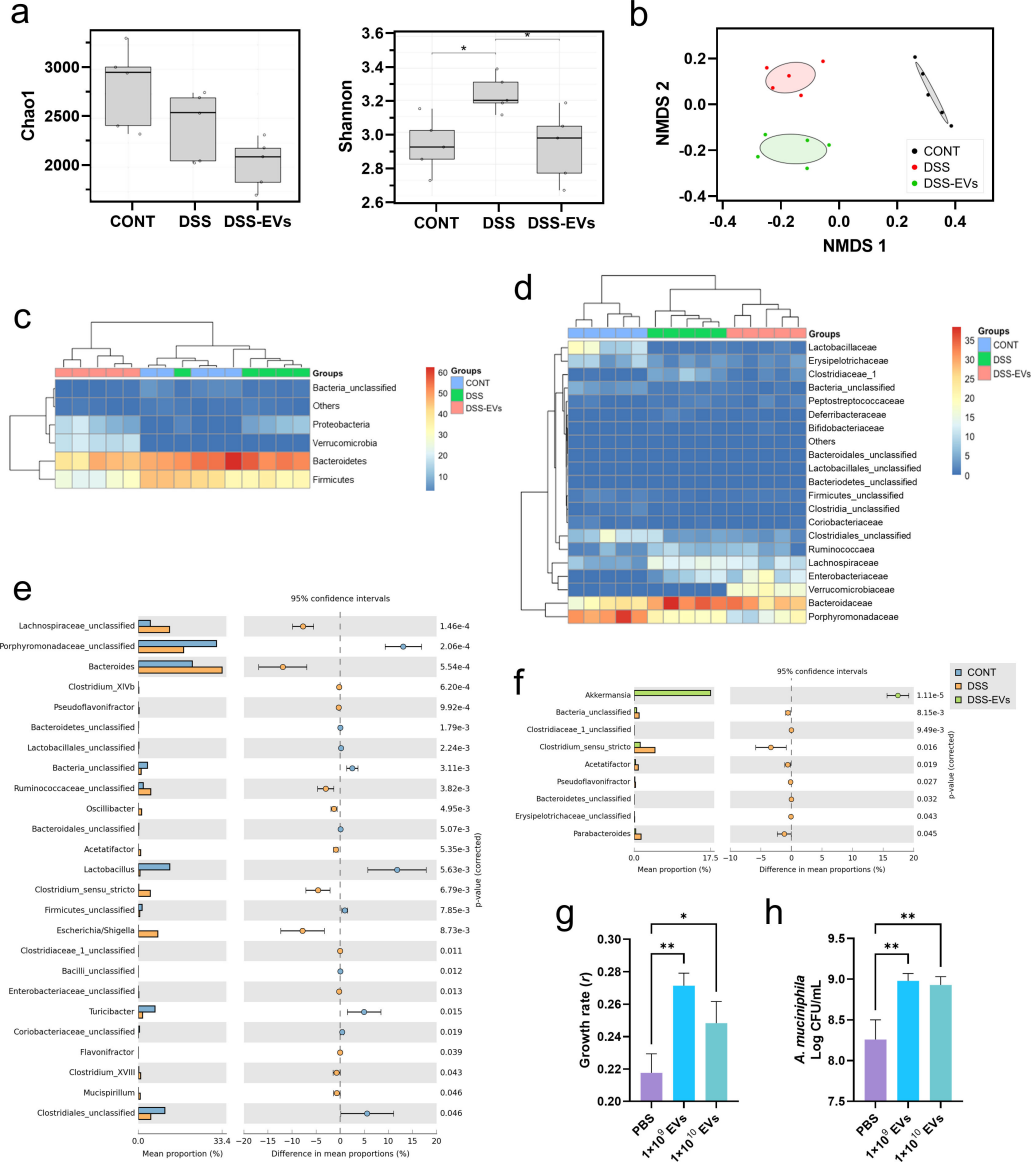
The fecal microbiota analysis shows a high abundance of *Akkermansia* sp*.* in EV-administered DSS-induced mice. (**a**) Comparison of ecological indices among groups. The distribution of species richness was measured using the Chao I index (left panel), and the distribution of species evenness was measured using the Shannon index (right panel). Boxes represent the interquartile ranges (IQRs) between the first and third quartiles (25th and 75th percentiles, respectively), and the horizontal line defines the medians. Whiskers represent the lowest and highest values within 1.5 times the IQR from the first and third quartiles, respectively. (**b**) Comparison of beta-diversity using the nonmetric multidimensional scaling analysis. Each dot in the figure denotes the microbiota profile of a single fecal sample, and colored dots denote each group. (**c and d**) Taxonomic composition of fecal microbiota at the phylum level (**c**) and the family level (**d**). (**e and f**) Analysis of differential abundance between CONT and DSS samples (**e**) and between DSS and DSS-EV samples (**f**). (**g and h**) *A. muciniphila* KCTC 15667 was cultured with PBS or EVs (1 × 10^9^ and 1 × 10^10^ particles/mL), and growth rate (**g**) and cell viability at the end point (**h**) were evaluated. Data represent the averages from five for metagenomics or six replicates for bacterial culture, and error bars represent standard deviations. Statistical analyses among the groups were performed using one-way ANOVA and Dunnett’s multiple comparison test. Growth rate of *A. muciniphila* was calculated using the growthcurver package of R. CONT, saline control; DSS, DSS-treated control; DSS-EVs, DSS induction with EV treatment.

Gut microbes play a critical role in maintaining host intestinal fitness and influencing the development of inflammatory diseases (24). We collected fecal samples from mice in the CONT, DSS, and DSS-EV groups on day 6 after the DSS treatment and characterized their microbiota composition using a metagenomic analysis to elucidate the effects of EVs on the gut microbiome in DSS-induced mice. A total of 5,225,598 sequenced reads (28,283–82,679 reads per sample) were analyzed based on read number normalization performed by randomly selecting 26,000 reads from each sample. A total of 2,204 operational taxonomic units (OTUs), with a range of 223 to 440 in each sample, were obtained using a 97% similarity level. The alpha diversity analysis shown in Fig. 3a indicates that the Chao1 index was not significantly changed by the DSS treatment. However, the Shannon index was increased by the DSS, while conditioning with EVs decreased the values to a level similar to that of the normal control. The nonmetric multidimensional scaling (NMDS) analysis also suggested that both treatments (i.e., DSS and EVs) significantly shifted the mouse gut microbiota (*P* < 0.05, Fig. 3b). The taxonomic classification analysis showed a significant increase in the phylum Verrucomicrobia in the DSS-EV group (Fig. 3c). At the family level, DSS treatment increased the abundance of the family Bacteroidaceae while decreasing the abundance of the families Porphyromonadaceae and Lactobacillaceae. EV treatment of DSS-treated mice increased the abundance of Verrucomicrobiaceae (Fig. 3d).

Differentially abundant genera in each group were further identified using STAMP analysis (Fig. 3e and f). DSS treatment significantly reduced the abundance of beneficial bacteria, such as *Lactobacillus* and *Turicibacter*, while increasing the abundance of *Escherichia*/*Shigella* and *Bacteroides*. DSS is known to induce colitis in mice by disrupting epithelial layers, allowing bacteria to infiltrate and ultimately inducing intestinal dysbiosis (25). On the other hand, treating mice with EVs increased the abundance of *Akkermansia* that resides on the mucus layer of gut epithelial cells, preserving the intestinal layers. Many studies have provided evidence of the beneficial roles of *Akkermansia* in alleviating colitis via reducing the expression of pro-inflammatory cytokines or expanding cytotoxic T lymphocytes in the colon (26, 27). To confirm the increase in *Akkermansia* in the gut microbiota due to EV treatment, *A. muciniphila* KCTC 15667 were incubated with two different doses of EVs (1 × 10^9^ and 1 × 10^10^ particles/mL) for 50 h, and the growth rate and viable cell number at the endpoint were measured. Consistent with metagenomic results, the growth rate of *A. muciniphila* was significantly increased in both high-dose and low-dose EV groups (Fig. 3g), and the end point viability was also significantly increased in both EV-treated groups (Fig. 3h). Collectively, these results suggest that the increase in *Akkermansia* abundance is involved in alleviating DSS-induced colitis.

### EV administration to DSS-induced mice increases the abundance of short-chain fatty acids in feces

**Fig 4:**
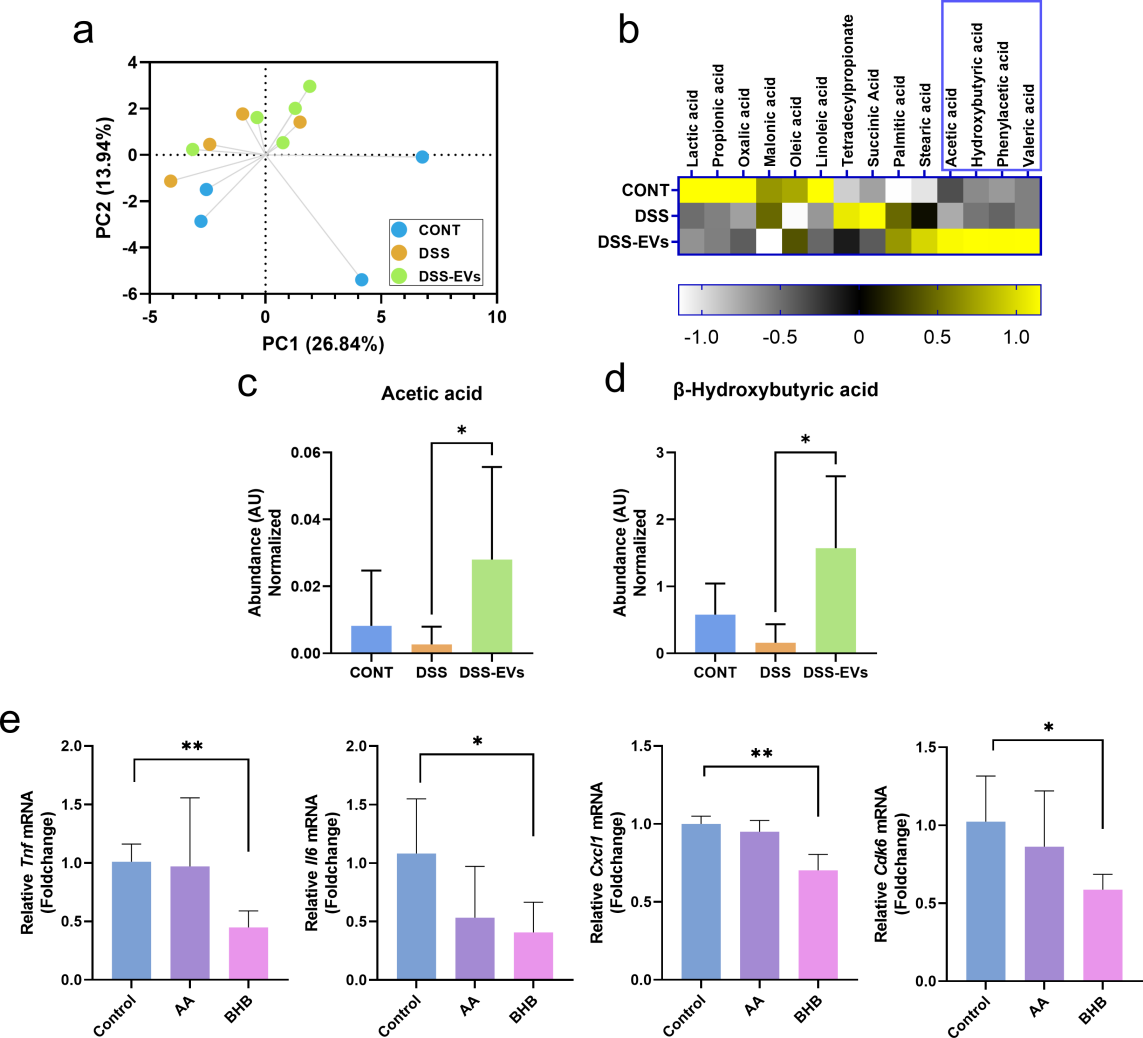
Administration of EV to DSS-induced mice increased the abundance of short-chain fatty acids in the feces, which reduced the expression of pro-inflammatory genes. (**a**) Principal component analysis of fecal metabolites. The intensities of metabolites were detected using GC-MS. (**b**) Heat map of changes in fatty acids and organic acids induced by EV treatment and DSS. Data are presented as unit variance scaled and utilized for the heat map plot. The blue box represents the most significantly increased metabolites in the DSS-EV group compared with the CONT and DSS groups. (**c and d**) Abundance of metabolites in mouse feces. Statistical analyses were performed using Student’s *t*-test, and an asterisk represents statistical significance at *P* < 0.05 (*). Data represent the averages from four to five replicates, and error bars represent standard deviations. (**e**) The expression of genes (*Tnf*, *Il6*, *Cxcl1*, and *Cdk6*) associated with the inflammatory response was analyzed in HT-29 human colorectal cancer cell lines treated with metabolites. Data represent the averages from four replicates, and error bars represent standard deviations. Statistical analyses of differences were performed using Student’s *t*-test. Asterisks represent statistical significance at *P* < 0.05 (*) and *P* < 0.01 (**). AA, acetate; BHB, β-hydroxybutyrate; CONT, saline control; DSS, DSS-treated control; DSS-EVs, DSS induction with EV treatment.

To further investigate how EV administration affected the metabolism of the host intestine and gut microbiota, we assessed the extent of the alterations in the mouse fecal metabolomic profiles of the three groups. Thirty fecal metabolites were identified, including amino acids, polyamines, organic acids, sugars, and free fatty acids. A multivariate analysis revealed slight metabolic differences among the groups, and EV administration resulted in distinct metabolic changes in the feces of DSS-treated mice (Fig. 4a). In particular, the metabolic alterations induced by EV treatment were prominently observed in organic acids and free fatty acids such as acetic acid and hydroxybutyrate (Fig. 4b through d). The increased abundance of these metabolites was only observed when EVs were administered to DSS-treated mice, implying a possible interaction between EV treatment and colitis induction associated with changes in the gut microbiota, mainly in the *Akkermansia* composition. Short-chain fatty acids produced by gut microbiota, such as *Akkermansia*, are known to possess immunomodulatory functions by activating signaling cascades that control immune functions (28). Our results also support the hypothesis that enhanced intestinal metabolites by *Akkermansia* regulate immune functions in mice with colitis treated with EV supplementation.

### Intestinal metabolites increased by *Akkermansia* reduce pro-inflammatory gene expression

Acetate is one of the representative short-chain fatty acids produced by *A. muciniphila* (29). The production of hydroxybutyrate has merely been reported, but a recent study showed that oral administration of *A. muciniphila* increases the intestinal concentration of the metabolite (30). In the current study, two metabolites, acetate and β-hydroxybutyrate (BHB), were added to HT-29 human colorectal cells, and pro-inflammatory gene expression was analyzed to verify the possible roles of intestinal metabolites in host immunomodulation. Consistent with findings from the present study, these metabolites reduced the expression of *Cdk6*, *Tnf*, *Il6*, and *Cxcl1* (Fig. 4e). Compared with acetate, BHB was significantly effective at suppressing gene expression. Based on these results, the supplementation of EVs in mice with colitis triggers the production of intestinal metabolites, BHB, possibly stimulated by the increase in *Akkermansia*, which are responsible for the attenuation of the inflammatory response in the host. Overall, our current study shows that bovine colostrum EVs protect the mice intestine from DSS-induced colitis through alterations in the gut microbiota and metabolite production, resulting in reduced pro-inflammatory response.

### *Akkermansia* and BHB enhanced by EVs prevents DSS-induced colitis via immune modulation

**Fig 5:**
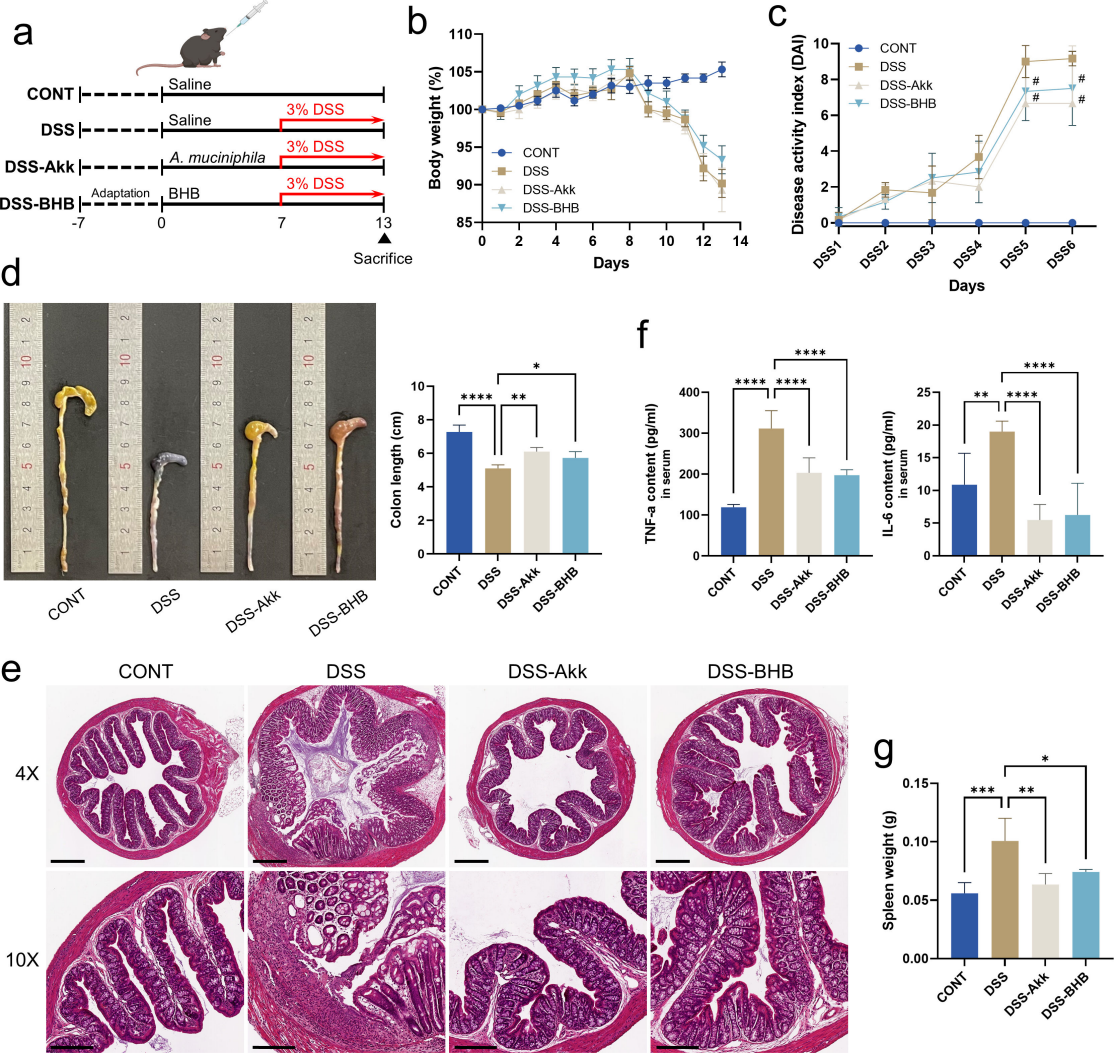
*Akkermansia* and BHB enhanced by EVs prevent DSS-induced colitis via immunomodulatory response. (a) *A. muciniphila* KCTC 15667 (10^8^ CFU/100 µL/mouse) and BHB (100 mg/kg) were administrated by oral gavage daily for 7 days, and subsequent colitis was induced by 3% DSS during the last 6 days concurrent with *A. muciniphila* and BHB treatment. (b) Body weight changes in mice during experiment. (c) Disease activity index. (d) Representative colons (left panel) and colon length (right panel). (e) Representative histological H&E staining of sections of the colon tissue. Scale bars in the upper panel represent 400 µM, and those in the lower panel represent 200 µM. (f) Serum levels of the pro-inflammatory cytokines TNF-α and IL-6. (g) Spleen weight. Data represent averages from six replicates, and error bars represent standard deviations. Statistical analyses comparing differences between the two groups were performed using Student’s *t*-test, and differences among the groups were performed using one-way ANOVA and Tukey’s multiple comparison test. Asterisks represent statistical significance at *P* < 0.1 (#), *P* < 0.05 (*), *P* < 0.01 (**), *P* < 0.001 (***), and *P* < 0.0001 (****). CONT, saline control; DSS, DSS-treated control; DSS-Akk, DSS induction with *A. muciniphila* treatment; DSS-BHB, DSS induction with β-hydroxybutyrate.

To verify whether the protective effect of EVs on colitis is due to the effects of EV-induced increased *Akkermansia* and intestinal metabolite BHB, DSS-induced colitis mice were treated with *A. muciniphila* and BHB and the extent of colitis was assessed. *A. muciniphila* KCTC 15667 (1 × 10^8^ CFU/100 µL/mouse) and BHB (100 mg/kg) were orally gavaged to mice for a total of 13 days, and to induce colitis, 3% DSS was given for 6 days (Fig. 5a). We confirmed that administration of *A. muciniphila* and BHB did not result in a significant difference in mouse body weight (Fig. 5b), but both *A. muciniphila* and BHB groups tend to decrease in DAI score (Fig. 5c) and showed a significantly recovered colon length (Fig. 5d) compared with the DSS group. Consistent with this, the histological analysis of colon tissues showed that the tissues disintegrated due to colitis were significantly less in *A. muciniphila* and BHB-treated colitis mice (Fig. 5e). In addition, to evaluate the anti-inflammatory role of *A. muciniphila* and BHB, we measured the levels of TNF-α and IL6 in mouse serum. The pro-inflammatory cytokines stimulated by DSS-induced colitis were significantly decreased in the *A. muciniphila* and BHB groups (Fig. 5f), and spleen weight, another inflammatory factor, was also significantly lower in the *A. muciniphila* and BHB groups than in the DSS group (Fig. 5g). Through these results, it was confirmed that *A. muciniphila* and BHB increased in the intestine by EV treatment could decrease the immune response and weaken the severity of colitis.

### bta-let-7a-5p prevents DSS-induced colitis via immunomodulation and regulation of the cell cycle

**Fig 6:**
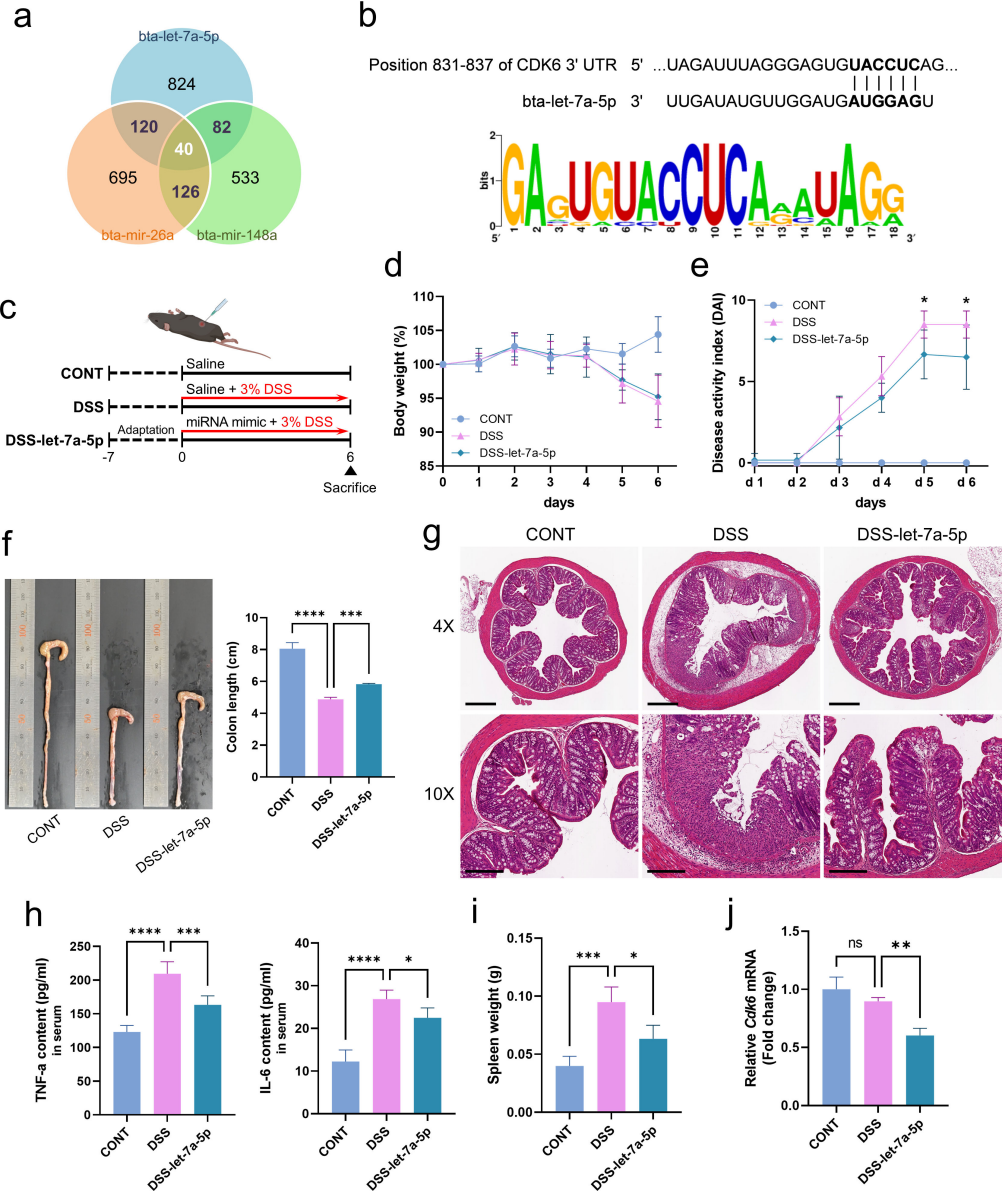
bta-let-7a-5p prevents DSS-induced colitis via immunomodulation and regulation of the cell cycle. (**a**) Venn diagrams showing the number of miRNA-mRNA interactions predicted by TargetScan. Mouse for three representative miRNAs present in milk EVs (bta-let-7a-5p, bta-mir-26a, and bta-mir-148a). (**b**) The *Cdk6* gene was one of the 40 shared mRNA targets, and a representative miRNA target binding site was predicted (upper panel). Conservation of the sequences among mammals was visualized using a Weblogo analysis (lower panel). (**c**) Schematic design of miRNA bta-let-7a-5p mimic treatment (5 nmole/mouse) by intraperitoneal injection with colitis induction by 3% DSS for 6 days. (**d**) Body weight changes. (**e**) Disease activity index. (**f**) Representative colons (left panel) and colon length (right panel). (**g**) Histological H&E staining of sections of the colon. Scale bars in the upper panel represent 400 µM and those in the lower panel represent 200 µM. (**h**) Serum levels of the pro-inflammatory cytokines TNF-α and IL-6. (**i**) Spleen weight. (**j**) The mRNA expression of *Cdk6*. Data represent averages from six replicates, and error bars represent standard deviations. Statistical analyses comparing differences between the two groups were performed using Student’s *t*-test, and differences among the groups were performed using one-way ANOVA and Tukey’s multiple comparison test. Asterisks represent statistical significance at *P* < 0.05 (*), *P* < 0.01 (**), *P* < 0.001 (***), and *P* < 0.0001 (****). CONT, saline control; DSS, DSS-treated control; DSS-let-7a-5p, DSS induction with bta-let-7a-5p mimic injection.

According to a previously published study by our research team, the intersections of each of the top 10 miRNAs identified in EVs isolated from bovine and human colostrum included let-7a-5p, miR-148a, and miR-26a (17). To further analyze whether miRNAs in bovine colostrum-derived EVs have a preventive effect on colitis, target genes of let-7a-5p, miR-26a, and miR-148a were predicted using the TargetScan database (31). As shown in Fig. 6a, the Venn diagram shows the numbers of overlapping miRNAs among the three major miRNAs, and the *Cdk6* gene was identified as a presumed common target of the miRNAs. Putative miRNA binding sites for miR bta-let-7a-5p in the mouse *Cdk6* gene sequence were predicted, and all binding sites had a good seed region and complementary base pairing in the 3’ end of the miRNAs that were highly conserved in mammals (Fig. 6b). Interestingly, *Cdk6* is widely known to regulate inflammatory and innate immune responses associated with the regulation of NF-κB-dependent transcription of inflammatory mediators (32, 33). Additionally, recent reports have provided insights into the role of CDK6 as a transcriptional regulator that links cell cycle progression to inflammation via the NF-κB signaling cascade.

Therefore, to determine whether bta-let-7a-5p could regulate cell cycle-related genes and has a preventive effect on colitis, bta-let-7a-5p mimic (5 nmole/mouse) were administered for 6 days to DSS-induced colitis mice by intraperitoneal injection and the severity of colitis and inflammatory markers was evaluated (Fig. 6c). bta-let-7a-5p mimic administration had no apparent effect on body weight recovery in DSS-induced colitis mice, but it was confirmed that the miRNA mimic significantly decreased the DAI score when compared with the DSS group (Fig. 6d and e). Consistent with this, the colon length shortened by colitis and disintegrated intestinal epithelial cells were also significantly restored upon bta-let-7a-5p mimic treatment (Fig. 6f and g). Serum pro-inflammatory cytokine levels were examined to investigate whether bta-let-7a-5p is involved in immunomodulation. In comparison to the DSS group, the levels of TNF-α and IL-6 were significantly lower in the DSS- bta-let-7a-5p group. The weight of the spleen was also found to be significantly lower than that in the DSS controls (Fig. 6h and i). In particular, to verify whether Cdk6, the target gene of bta-let-7a-5p, is inhibited, the expression level of Cdk6 in the intestinal tissue of the mouse was measured and it was confirmed that mRNA expression was significantly lower in the miRNA mimic-treated group (Fig. 6j). These findings imply that the bta-let-7a-5p derived from bovine colostrum EVs target cell cycle regulators associated with regulating the immune response, resulting in attenuation of inflammatory responses in mice with colitis.

## DISCUSSION

**Fig 7:**
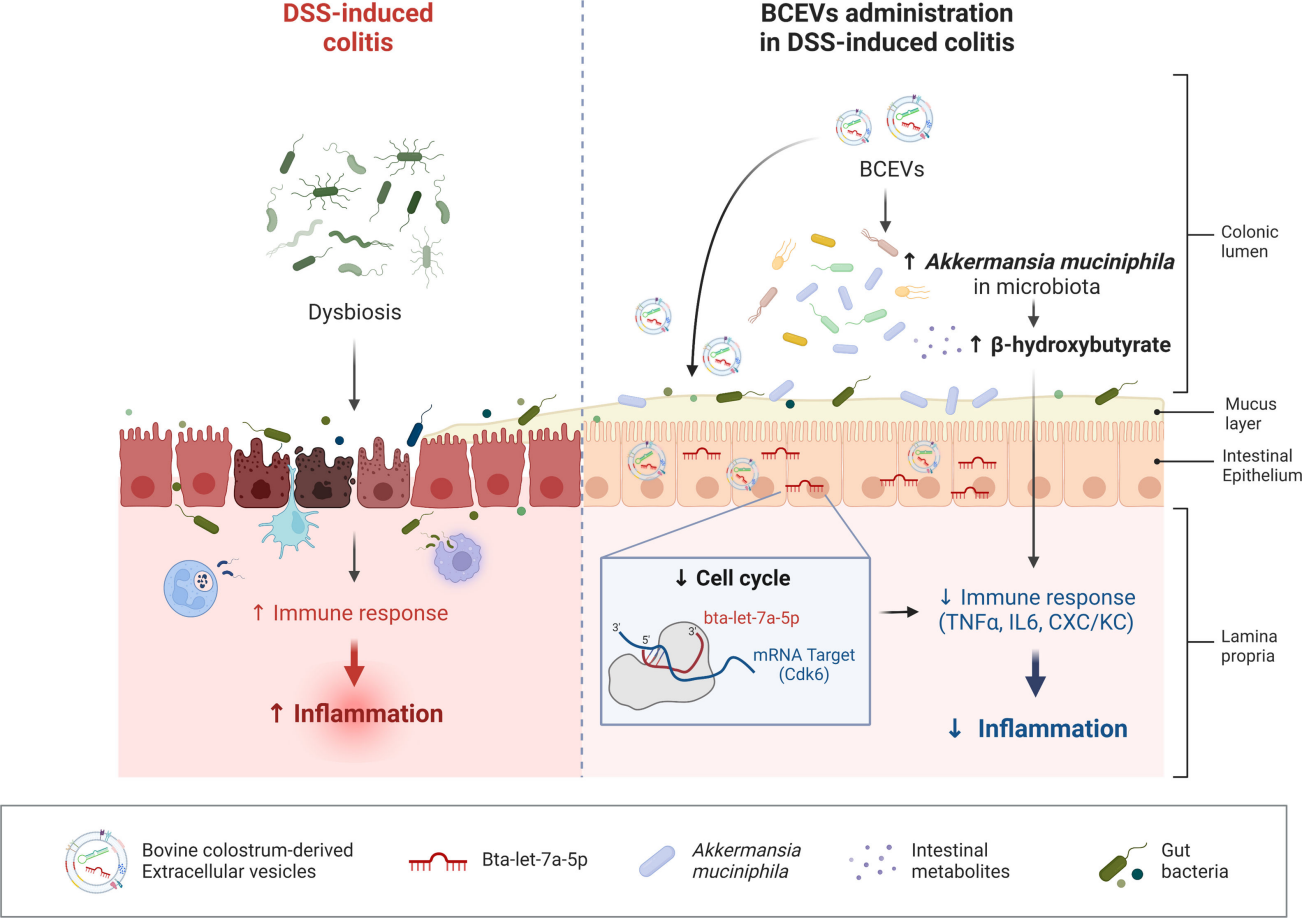
Schematic diagram. Schematic showing the results of integrated transcriptomic and metagenomic analyses of the roles of bovine colostrum EVs in host systemic immune regulation. Bovine colostrum EVs and bta-let-7a-5p protected the intestine from DSS-induced colitis by decreasing cell cycle regulator-mediated pro-inflammatory processes. This protective effect is also mediated by alterations in the gut microbiota (*Akkermansia* genus) and metabolite production (β-hydroxybutyrate).

Based on accumulating evidence, EVs play important roles in numerous physiological processes, including immune regulation (34). Although many studies have been published related to host mammal-derived EVs, EVs from dietary sources are attracting increasing attention due to their relevance in modulating cellular processes (35). In the present study, we observed alleviated inflammation in a mouse DSS-induced colitis model following an EV treatment and investigated the possible mechanisms of the effects using multiomic approaches, including metagenomics, metabolomics, and transcriptomics. Transcriptomic analyses suggested a possible immunoregulatory mechanism via the inflammatory response mediated by the cell cycle regulator CDK6. The administration of EVs exerted a substantial effect on altering gut microbiota composition, especially increasing the abundance of *Akkermansia* in mice with colitis. The fecal metabolomics analysis indicated increased levels of short-chain fatty acids and relevant organic acids in accordance with changes in the microbial composition. Additional *in vivo* experiments demonstrated that BHB and *Akkermansia*, which are increased by the administration of EVs, are involved in alleviating colitis via regulating the immune system. Moreover, the microRNA bta-let-7a-5p, which is most prevalent in EVs of bovine colostrum, attenuated the symptoms of colitis via regulating the target gene Cdk6 and modulating the intestinal immunity of mice, suggesting that the possible immunomodulatory effects of bovine colostrum EVs and its miRNA were linked to the alteration in the gut microbial composition and immune-related gene regulation (Fig. 7).

The hypothesis that milk EVs prevent intestinal inflammation has been supported by numerous studies using different sources of milk from humans (36), cows (37), pigs (7), camels (38), and rats (39). Researchers applied these materials to genetic mouse models, such as *Mdr1a^−/−^* and *IL-1Ra^−/−^* mice, and inflammation-induced mice, or *in vitro* cell lines to identify the effects of EVs on inflammation. Overall, milk EVs reduced cecal inflammation (40), prevented intestinal damage (36), exerted anticancer effects (38), and promoted intestinal stem cell proliferation (39). Isolated human milk EVs decreased inflammation, mucosal injury, and mucus production (36). In addition, Xie et al. found that overexpression of miRNAs (miR-4334, miR-219, and miR-338) present in porcine milk EVs in IPEC-J2 cells inhibited TLR4/NF-κB and P53 pathways, resulting in the attenuation of LPS-induced apoptosis (7). Consistent with previous findings, our current results clearly showed that bovine colostrum EVs attenuated DSS-induced colitis in mice through immunomodulation and through cell cycle regulation.

Emerging evidence has revealed that EVs contribute to the regulation of the cell cycle (41 – 45). These EVs were mostly isolated from the serum of patients with cancer, cancer cell lines, or bovine granulosa cells. The exosomal association with cell cycle regulation affects arrest in G0/G1 phase, inhibiting proliferation via IFN-α (44), NRF2 (43), or protein tyrosine kinases (41), cross-talking with immunosuppressive microRNAs. However, unlike cellular EVs, studies have not been published on the regulatory effects of exosomes derived from dietary sources on the cell cycle. In this study, we documented that the expression of genes involved in cell cycle regulation was downregulated in the intestine of mice with colitis upon the administration of EVs through the suppression of CDK6/NF-κB signaling via transcriptomic analysis. Intestinal epithelial cells play an important role in maintaining immune response and homeostasis to gut microorganisms and foreign factors. The excessive proliferation of intestinal epithelial cells may lead to autoimmune diseases, disrupt homeostasis, and fail to maintain the intestinal barrier (46, 47). On the other hand, treatment of normal mice with milk EVs did not alter gene expression (Fig. S1), implying that EVs selectively affect target recipient cells depending on the type and status of the cells. Recently, Sancho-Albero et al. reported the selectivity of *in vitro* EV transfer to different cell types, including cancer, metastatic, stem, or immunological cells (48). Based on these findings, the targeting specificity of bovine colostrum EVs remains to be understood, and an effective strategy for maximizing the beneficial effects of the particles on host health must be developed.

EVs are involved in cell-cell signaling and possess the potential to modulate host-microbe interactions (12). In the present study, we detected a marked increase in Verrucomicrobia, specifically *Akkermansia*, in DSS-treated mice administered EVs. Recently, Zhou et al. reported that dietary exosomes (similar cellular particles to EVs) in bovine milk alter bacterial communities in the murine cecum (49). At the family level, the abundance of Verrucomicrobiaceae was reduced in exosome/RNA-sufficient diets compared with exosome/RNA-depleted mice, corroborating the hypothesis that bovine milk EVs would increase the abundance of Verrucomicrobiaceae, which is consistent with our current results.

The intestinal mucous layer plays a protective role in the host and is the site for many host-microbe interactions. The mucin-degrading bacterium *Akkermansia* uses mucus as a nutrient source not only to degrade mucins but also to stimulate mucin synthesis as an autocatalytic process (50). Previous studies suggested that mucin-utilizing species such as *Akkermansia* protect the host from intestinal pathogens and aid in microbiota restoration (50, 51). The expression profile of *Muc2* and *Muc3* documented in this study is consistent with previous reports showing that these mucins are the main genes expressed in the intestinal mucosa, and their expression decreases in a patient with inflammatory diseases (52). Since the integrity of the mucus layer is critical for gut health, MUC2, which encodes a secretory mucin protein, is regarded as the main protector against inflammation and penetration of commensal bacteria (53). Unlike MUC2, cell surface-linked Mucin 1 (encoded by *Muc1*) is often associated with tumor-induced changes in host immunity (54). It also increases epithelial inflammation in response to inflammatory stimuli, resulting in increased expression in the DSS group (55). However, although an earlier study reported that *Muc4* is downregulated in humans suffering from IBD, our current result revealed its overexpression only in the DSS-EV group; thus, further investigation on the effects of bovine colostrum EVs on MUC4-associated mucin production is required (56).

*Akkermansia* is known to produce short-chain fatty acids such as acetate and propionate (50). Our metabolomic analysis indicated increased levels of some short-chain fatty acids and relevant organic acids upon EV treatment, along with the increase in the *Akkermansia* composition. Previous studies have reported that the levels of propionate in the intestine were associated with the relative abundance of *Akkermansia* and inhibition of mucin utilization (57). In this study, we found that the relative abundance of *Akkermansia* increased in the DSS-EV group compared with the CON and DSS groups, while intestinal propionate levels were elevated in the CON group compared with the DSS and DSS-EV groups. *A. muciniphila* and propionate may play an important role in gut health and metabolic disease prevention, and this relationship is increasingly being explored. However, there remains to be further study on the complexity of this relationship and its interaction with other factors in the body.

*In vitro* treatment of human cell cultures with acetate and BHB and *in vivo* validation with *A. muciniphila* and BHB supported the hypothesis that *Akkermansia* and intestinal metabolites enhanced by *Akkermansia* induce immunomodulatory responses in the host. Acetate is the most abundant short-chain fatty acid in the gut and is produced from acetyl-CoA. The acetate/GPR43 pathway stimulates potassium efflux and hyperpolarization in colonocytes, controlling inflammation and promoting epithelial repair in the colon (58). Hydroxybutyrate is a four-carbon organic acid as a derivative of butyric acid. It has three types of isomers distinguished by the location of a hydroxyl functional group. 3-Hydroxybutyrate, also known as β-hydroxybutyrate, is the most well-characterized molecule, which is relevant to fatty acid oxidation and immune responses in human (59). According to Youm et al., BHB has been shown to inhibit NLRP3 inflammasome-mediated interleukin (IL)-1β and IL-18 in human cells and *in vivo* (60). In addition, BHB suppresses inflammasome and endoplasmic reticulum stress through AMP-activated protein kinase (AMPK) activation and forkhead O bax transcription factor 3 (FOXO3) signaling (61). In this study, oral administration of exogenous BHB showed anti-inflammatory effects on colitis, which was consistent with the previous study that the damaged intestine was restored by the increase of M2 macrophage polarization caused by the administration of BHB (62). Interestingly, a recent study has shown that oral administration of *A. muciniphila* elevated the intestinal concentrations of hydroxybutyrate (30). The result corroborates our result on the immunomodulatory effect of the BHB, possibly induced by the increase of *Akkermansia* with the EV supplement.

The existence of numerous miRNAs in bovine colostrum EVs and that they are absorbed into the body and affect target genes have been reported (63, 64). In this study, exogenous let-7a-5p was shown to alleviate colitis through inflammatory response and cell cycle-related Cdk6 gene regulation. Similar to our findings, Zhang et al. showed that let-7a-5p modulates IL-6 expression via the Ras-MAPK pathway in chronic rhinosinusitis with nasal polyps (65). Interestingly, let-7a-5p was found to be downregulated in colorectal cancer (66), and let-7a-5p increased colorectal cancer cell apoptosis by regulating CREB5 and inhibited colon cancer cell proliferation, migration, and metastasis (19). The G1 cell cycle kinase CDK6 regulates cell cycle progression and is implicated in a number of cancer types. Recently, CDK6 appears to have versatile functions; in addition to being a cell cycle regulator, it functions in transcriptional regulation, differentiation, and inflammation (33). CDK6 is present and/or binds several promoters, including cyclin-dependent kinase inhibitor 2A (p16INK4a) and vascular endothelial growth factor (VEGF-A), which induce growth inhibition and angiogenesis (67). In addition, CDK6 recruits the NF-κB subunit p65 to specific genes specifically involved in inflammation (32). Upon RNAi-mediated suppression of *CDK6*, suppressed activation of NF-κB further downregulated TNF-induced gene expression (68).

In conclusion, bovine colostrum EVs and bta-let-7a-5p protected the intestine from DSS-induced colitis by decreasing cell cycle regulator-mediated pro-inflammatory processes. This protective effect is also mediated by alterations in the gut microbiota and metabolite production. Our current findings provide insights into the encouraging benefits of dietary source-derived EVs and miRNAs on organic acid production and ultimately stimulating gut health in humans, suggesting that supplementation of dietary colostrum EVs and miRNAs is a novel preventive strategy for the treatment of IBD.

## MATERIALS AND METHODS

### Isolation and characterization of EVs from bovine colostrum

EVs were isolated from bovine colostrum using a previously developed method in our group to maximize the yield of microvesicular RNA (63). The established method met the Minimal Information for Studies of Extracellular Vesicles (MISEV) guidelines, verifying their purity by ratio of RNA and protein and the presence of a biomarker CD9 (63). Briefly, 10 mL of colostrum was centrifuged at 1,200 × *g* for 10 min to defat the colostrum and then at 16,000 × *g* for 1 hour to pellet cell debris. The sample was further centrifuged at 50,000 × *g* for 1 hour to generate the whey fraction. The whey fraction was then ultracentrifuged at 100,000 × *g*, followed by ultracentrifugation of the supernatant at 120,000 × *g* for 90 min to pellet extracellular vesicles. The extracellular vesicles were then resuspended in PBS and frozen (−80°C) until use. The average size and particle concentration of the isolated EVs were measured by nanoparticle tracking analysis (Malvern NanoSight NS300, Malvern Technologies, Malvern, UK).

### *Akkermansia muciniphila* culture

*A. muciniphila* strain KCTC 15667 were cultured in brain heart infusion (BHI) broth (BD Difco, USA) with 0.5% mucin type II (Sigma, St. Louis, USA) at the anaerobic chamber (COY Laboratories, Grass Lake, USA). After anaerobic incubation at 37°C for 2 days, the supernatant was removed by centrifugation, and the resulting bacterial pellet was washed twice with anaerobic PBS and then suspended in anaerobic buffer to a density of 1 × 10^9^ CFU/mL and immediately used for oral inoculation. To evaluate the growth curves of *A. muciniphila* supplemented with EVs, two different doses of EVs (1 × 10^9^ and 1 × 10^10^ particles/mL) were added to BHI broth, and then, *A. muciniphila* was inoculated. The absorbance at OD_600_ of bacterial culture was measuring every 2 hours for 2 days, and the number of viable cells was measured at the end point.

### Animals

All animal experiments were performed after review and approval by the Institutional Animal Care and Use Committee of Seoul National University (SNU-210105-4-4). Eight-week-old female mice (C57BL/6) were purchased from Orient-Bio Inc. (Seungnam, Republic of Korea) and were housed under constant conditions (12 h light/dark cycle, 55% ± 5% humidity, and 22°C ± 1°C). Mice were provided water and food *ad libitum* throughout the experimental period.

### Induction of colitis in mice and treatment

Female mice (C57BL/6) per group (*n* = 6–10) were housed per individually ventilated cage with bedding changed weekly. To induce colitis, mice were administered water containing 3% (wt/vol) DSS (36–50 kD; MP Biomedicals, LLC, IIkirch, France) *ad libitum* for the last 6 days of each experiment. For DAI scoring, body weight, stool consistency, and rectal bleeding were monitored every day.

For treatment of EVs, mice were randomly allocated into four groups: CONT (saline control), DSS (DSS-treated control), DSS-EVs (DSS induction with EV treatment), and CONT-EVs (EV treatment without DSS treatment). Bovine colostrum EVs (1 × 10^11^ particles/mouse) were administered orally by gavage to the CONT-EV and DSS-EV groups daily for 4 weeks, while the control groups of CONT and DSS were treated with saline. *A. muciniphila* KCTC 15667 (1 × 10^8^ CFU/100 µL/mouse) and BHB (100 mg/kg; Sigma, St. Louis, USA) were orally administrated daily for 13 days, and bta-let-7a-5p (5 nmole/mouse; Bioneer, Daejeon, Korea) were intraperitoneally injected daily for 6 days at the same time as colitis induction.

### Collection of tissues

The abdominal cavity was incised, and the spleen, large intestines, and small intestines were collected. The distance between the most distal part of the rectum and the ileocecal valve was measured to determine the colon length. For RNA extraction and the quantification of the mRNA using RT-PCR, the tissue was cleansed with PBS to remove any gut contents present and frozen (−80°C) until use. The middle part of colon tissues was fixed with paraformaldehyde for the histological examination using hematoxylin and eosin staining.

### Quantification of serum cytokine and intestinal mRNA levels

Levels of selected cytokines were measured in serum. The serum samples were analyzed using a Magnetic Luminex Human Premixed Multianalyte Kit (R&D Systems Inc., Minneapolis, MN), and all procedures were performed in strict accordance with the manufacturer’s protocol. Real-time quantitative PCR was used to quantify mRNAs. RNA was extracted using an RNAeasy Kit (QIAGEN) according to the manufacturer’s protocol. The extracted RNA was quantified, and its purity was assessed using a NanoDrop spectrophotometer (Optizen-NanoQ, Mecasys Co. Ltd., Korea). The cDNA templates were generated by reverse transcription of RNA using a miScript II Reverse Transcription Kit (QIAGEN) and a SimpliAmp Thermal Cycler (Applied Biosystems, USA). RT-qPCR was then performed using the obtained cDNAs in a StepOne Plus Real Time PCR System (Applied Biosystems, USA). The miScript SYBR Green PCR Kit was used for RT-qPCR. The primer sequences used for RT-PCR are shown in Table S1.

### Transcriptome analysis using RNA sequencing

Total RNA was extracted using TRIzol reagent (Invitrogen, USA) and a RNeasy Mini kit (QIAGEN) according to the manufacturer’s instructions. The concentration (260/280 ratio and 260/230 ratio) and quality of total RNA were determined using a spectrophotometer (SpectraMax ABS Plus, Molecular Devices, San Jose, CA). Two pooled replicates from mice intestinal RNA samples were used for transcriptomic analysis. For RNA-seq, TruSeq RNA Sample Prep Kit v2 (Illumina, San Diego, CA, USA) was used according to the manual, and the cDNA library was generated according to the basic protocol provided by Illumina. Libraries were then sequenced on an Illumina HiSeq 2000 platform with paired-end read sequencing (2 × 150 bp).

The raw reads were used to remove the adapter sequence using Trimmomatic 0.38 (69); bases with a base quality less than 3 were removed from the ends of the reads, and bases were removed if the window size = 4 and mean quality = 15 were not satisfied with the sliding window trim technique. Afterwards, trimmed data were generated by removing reads shorter than a minimum length = 36 bp, and further analysis was performed based on high-level quality reads through this quality control procedure. The index of the reference genome was constructed using the Hisat2 v2.1.0 program (70), and paired-end clean reads of *Mus musculus* were read and compared. Next, uniquely mapped reads were quantified using Subread/featureCounts version v1.5.1 (71) with ENSEMBL version 82 transcriptome definitions. The generated data were subjected to a differential expression analysis between various types of samples by applying the R package edgeR (72), and the threshold values |log2 fold change > 1| and *P* < 0.05 were used in this study to define genes that were significantly differentially expressed.

Gene Ontology annotations were analyzed using the DAVID online tool (73) and clusterProfiler (74) and visualized using ClueGO (75) to identify the functions of differentially expressed genes. The analysis was performed on selected DEGs with a focus on gene functional annotations of cellular components (CC), biological processes (BP), and molecular functions (MF). The Kyoto Encyclopedia of Genes and Genomes (KEGG) pathway assignments and enrichment analysis were conducted using the Search Tool for the Retrieval of Interacting Genes/Proteins v11.0 (STRING) (76) with an FDR-adjusted *P* ≤ 0.05. Sequence data used in this study were deposited to the Gene Expression Omnibus repository with the project number GSE178663 (https://www.ncbi.nlm.nih.gov/geo).

### Fecal microbiome analysis using 16S deep sequencing

Feces were collected on day 6 after DSS treatment from each individual mouse. Fecal DNA was extracted using the PowerFecal DNA Isolation Kit (QIAGEN, Hilden, Germany). The V4 region of the 16S rRNA gene amplified by PCR was subsequently sequenced using MiSeq (Illumina Inc., San Diego, CA, USA) at Macrogen Inc., Korea, according to the manufacturer’s instructions. Sequence data were processed using mothur (77) according to the protocol described in MiSeq SOP (https://mothur.org/wiki/miseq_sop/). Briefly, paired-end assembly was performed using the make.contig mothur subroutine, and reads with ambiguous base calls shorter than 250 bp or longer than 350 bp were removed using the screen.seqs subroutine. Reads were aligned to the Silva rRNA gene database version 138. Machinery sequencing errors were corrected using the pre.cluster subroutine. Taxonomic classification was performed based on RDP database version training set 18 with the classify.seqs mothur subroutine. Mitochondrial and cyanobacterial sequences were excluded from further analysis. The number of reads was normalized by randomly taking a specific number of reads using the sub.sample mothur subroutine. Operational taxonomic units were assigned using the opti.clust mothur subroutine at 3% dissimilarity. The nonmetric multidimensional scaling analysis was performed using an nmds mothur subroutine. Functional profiling of the microbial community was predicted using PICRUSt (78). Sequence data used in this study were deposited to the Short Read Archives with the project number PRJNA738604.

### Fecal metabolome analysis

The fecal samples were collected and stored at −80°C until the metabolomic analysis. Each fecal sample was weighed, diluted in methanol to a final concentration of 20 mg/mL, and vortexed for 5 min on ice. After centrifugation at 15,000 × *g* for 5 min at 4°C, the upper layer of the supernatant was filtered with 0.2-µM pore size polyvinylidene fluoride (PVDF) syringe filters (Whatman, Maidstone, England). Aliquots of 200 µL of the filtered supernatant were concentrated to dryness in a vacuum concentrator and stored at −80°C prior to derivatization and analysis using GC-MS.

The extract was derivatized with 30 µL of a solution of 20 mg/mL methoxyamine hydrochloride in pyridine (Sigma, St. Louis, USA) at 30°C for 90 min, and 50 µL of N,O-bis(trimethylsilyl)trifluoroacetamide (BSTFA; Sigma, St. Louis, USA) was subsequently added and incubated at 60°C for 30 min. Fluoranthene was added to the extract as an internal standard. The GC-MS analysis was conducted using a Thermo Trace 1310 GC (Waltham, MA, USA) coupled to a Thermo ISQ LT Single Quadrupole Mass Spectrometer (Waltham, MA, USA). A DB-5MS column with a 60-m length, 0.2-mM i.d., and 0.25-µM film thickness (Agilent, Santa Clara, CA, USA) was used for separation. For analysis, the sample was injected at 300°C and a split ratio of 1:5 with 7.5 mL/min helium as the split flow gas. The metabolites were separated with 1.5 mL of constant flow helium and an oven ramp of 50°C (2 min hold) to 180°C (8 min hold) at 5°C/min, to 210°C at 2.5°C/min, and to 325°C (10 min hold) at 5°C/min. The mass spectra were acquired in a scan range of 35–650 m/z at an acquisition rate of 5 spectra per sec. The ionization mode was subjected to electron impact, and the temperature for the ion source was set to 270°C. The spectra were processed with Thermo Xcalibur software using automated peak detection, and the metabolites were identified by matching the mass spectra and retention indices using the NIST Mass spectral search program (version 2.0, Gaithersburg, MD, USA). The metabolite data were then normalized based on the intensity of the fluoranthene internal standard.

### Cell culture and qRT-PCR of pro-inflammatory genes

The human colon adenocarcinoma cell line HT-29 was routinely grown in RPMI 1640 medium (HyClone, Logan, UT, USA) containing 4.5 g of glucose per liter and supplemented with 10% fetal calf serum (Welgene, Gyeongsan-si, Korea). For the evaluation of metabolite effects on host gene expression, HT-29 cells (1 × 10^5^ cells/well) were seeded in 6-well plates and incubated for 48 h. After confirming formation of the monolayer, the culture media were discarded, and 100 µL of media containing 1 mM acetic acid (Sigma, St. Louis, USA) and BHB (Sigma, St. Louis, USA) were added to the cells and incubated for 24 h. After incubation, the cells were harvested, and RNA was extracted followed by cDNA synthesis and quantitative PCR as described above. The primer sequences used for RT-PCR are shown in Table S1.

### miRNA target gene prediction

The number of miRNA-mRNA interactions and a representative miRNA target binding site were predicted by TargetScan database (version 7.2). Conservation of the sequences among mammals was visualized using a Weblogo analysis.

### Statistical analysis

Significant differences in microbial communities between groups were examined using analysis of molecular variances (AMOVA) with an amova mothur subroutine. STAMP (statistical analysis of taxonomic and functional profiles) was used to conduct the principal component analysis and Welch’s *t*-test (79). ALDEx2 (80) was used to select significantly enriched or depleted metabolites from the predicted metabolic activities with PICRUSt2. Growth rates of *A. muciniphila* were analyzed using the Growthcurver R package (81). The comparison between the two groups is performed using Student’s *t*-test, and one-way analysis of variance (ANOVA) was used to compare among different treatment groups, and each group was compared with the other using Tukey’s test to assess any differences from the control and between the treatments, unless otherwise indicated. Statistical analyses and visualization were conducted using GraphPad Prism 9.0 (San Diego, CA, USA).

## Data Availability

All data needed to evaluate the conclusions in the paper are present in the paper and/or the supplemental material. Additional data are available from the authors upon request.

## SUPPLEMENTAL MATERIAL

Supplemental material spectrum.00121-23-s0001.docx Tables S1 and S2, Fig. S1, and legend for Movie S1.

Movie S1A and B spectrum.00121-23-s0002.mp4 Restored inflammation-associated mouse activity between DSS (A) and DSS-EVs (B) groups.

ASM does not own the copyrights to Supplemental Material that may be linked to, or accessed through, an article. The authors have granted ASM a non-exclusive, world-wide license to publish the Supplemental Material files. Please contact the corresponding author directly for reuse.
