# Supplementary material for: Alleviation of DSS-induced colitis via bovine colostrum-derived extracellular vesicles with microRNA let-7a-5p is mediated by regulating Akkermansia and β-hydroxybutyrate in gut environments
Source: Microbiol Spectr. 2023 Nov 15;11(6):e00121-23. doi: 10.1128/spectrum.00121-23 (PMC10714758; doi:10.1128/spectrum.00121-23)
Supplement: Supplemental material — Tables S1 and S2, Fig. S1, and legend for Movie S1. [file spectrum.00121-23-s0001.docx]

**SUPPORTING INFORMATION**

**Alleviation of DSS-induced colitis via Bovine colostrum-derived extracellular vesicles with microRNA *let-7a-5p* is mediated by regulating *Akkermansia* and β-hydroxybutyrate in gut environments**

Daye Mun^a†^, Minkyoung Kang^b†^, Minhye Shin^c†^, Hye Jin Choi^a^, An Na Kang^a^, Sangdon Ryu^a^, Tatsuya Unno^d^, Brighton E. Maburutse^e^, Sangnam Oh^b*^, and Younghoon Kim^a*^

^a^Department of Agricultural Biotechnology and Research Institute of Agriculture and Life Science, Seoul National University, Seoul 08826, Korea

^b^Department of Functional Food and Biotechnology, Jeonju University, Jeonju, 55069, Korea

^c^Department of Microbiology, College of Medicine, Inha University, Incheon 22212, Korea

^d^Subtropical/tropical Organism Gene Bank and Faculty of Biotechnology, Jeju National University, Jeju 63243, Korea

^e^Department of Animal Production Sciences, Marondera University of Agricultural Sciences & Technology, P.O. Box 35, Marondera, Zimbabwe

^†^These authors contributed equally to this work.

*To whom correspondence should be addressed: osangnam@jj.ac.kr and ykeys2584@snu.ac.kr

**Table S1.** Primers used in this study for qRT-PCR

| Primer | Sequence (5’-3’) |
| --- | --- |
| mouse Muc2 | Forward: TCAGCACACCAACCAAAACC  Reverse: CACTTCAGCGGCACAATCTC |
| mouse Muc3 | Forward: CCGGACCTCAATGACAACACT  Reverse: ACCACGATGCTGCCATTCCT |
| mouse Muc1 | Forward: AGTGCCTCTGACGTGAAGTCAC  Reverse: GGGAGGGAACTGCATCTCATTC |
| mouse Muc4 | Forward: AAGCGTGTTGCCTCTTCACCTG  Reverse: AGGTGGCTGAGACTGCTGTGTT |
| mouse Cdk6 | Forward: GCTTCGTGGCTCTGAAGCGCG  Reverse: TGGTTTCTGTGGGTACGCCGG |
| mouse Sac3d1 | Forward: CAACTTGGGTTCTGTGGAAGC  Reverse: GGTAGGGTAAGGTGCGAAGC |
| mouse Ncapd3 | Forward: GCAGAGTATCGGACCTACGC  Reverse: CCCGGTGTGGAATCTTGGAA |
| mouse Gapdh | Forward: CATCACTGCCACCCAGAAGACTG  Reverse: ATGCCAGTGAGCTTCCCGTTCAG |
| human Tnf | Forward: CCAGGGACCTCTCTCTAATCAGC  Reverse: CTCAGCTTGAGGGTTTGCTACAA |
| human Il6 | Forward: AGACAGCCACTCACCTCTTCAG  Reverse: TTCTGCCAGTGCCTCTTTGCTG |
| human Cxcl1 | Forward: AGATTCTATGTTAATATTTTAGGTGTAAAATAAT  Reverse: AACTAACTTGGGGTTGACATTTC |
| human Cdk6 | Forward: CAACTAGGAAAAATCTTGGACGTGAT  Reverse: TTGGTTGGGCAGATTTTGAAT |
| human Gapdh | Forward: TGCACCACCAACTGCTTAGC  Reverse: GGCATGGACTGTGGTCATGAG |

**Table S2**. Top 10 most significant DE genes

| **DSS *vs.* CONT** | | | **DSS-EVs *vs.* DSS** | | |
| --- | --- | --- | --- | --- | --- |
| Gene | Full name | Change in DSS | Gene | Full name | Change in DSS-EVs |
| *S100a9* | S100 calcium binding protein A9 | Up | *Sac3d1* | SAC3 domain containing 1 | Down |
| *S100a8* | S100 calcium binding protein A8 | Up | *Lats1* | Large tumor suppressor kinase 1 | Down |
| *Cd300lf* | CD300 molecule like family member f | Up | *Ahctf1* | AT-hook containing transcription factor 1 | Down |
| *Ifitm1* | Interferon induced transmembrane protein 1 | Up | *Clspn* | Claspin | Down |
| *Lbp* | Lipopolysaccharide binding protein | Up | *Gspt2* | G1 to S phase transition 2 | Down |
| *Plgrkt* | Plasminogen receptor with a C-terminal lysine | Down | *Sgol2a* | Shugoshin 2A | Down |
| *Ccl5* | C-C motif chemokine ligand 5 | Up | *Ncapd3* | Non-SMC condensing II complex subunit D3 | Down |
| *Ccl19* | C-C motif chemokine ligand 19 | Up | *Dsn1* | MIS12 kinetochore complex component | Down |
| *Havcr2* | Hepatitis A virus cellular receptor 2 | Up | *Steap3* | STEAP family member 3 | Down |
| *Ltb4r1* | Leukotriene B4 receptor 1 | Up | *Cdk6* | Cyclin-dependent kinase 6 | Down |


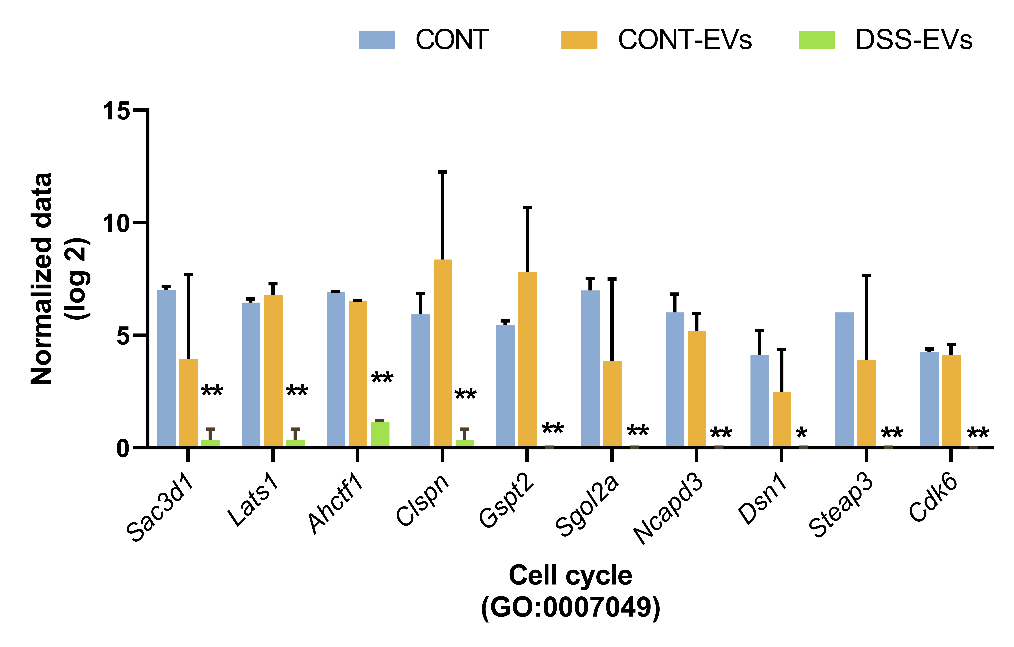


**Fig. S1.** **Cell cycle-associated gene expression with EV treatment in normal mice.** The gene expressions, downregulated in DSS-EVs group, were not affected by the EV treatment.

**Supplementary Movie S1.**

Restored inflammation-associated mice activity between DSS (A) and DSS-EVs (B) groups. For EVs treatment, bovine colostrum EVs (1×10^11^ particles/mouse) were administered orally by gavage daily for three weeks, and colitis was induced in mice using 3% DSS and with for 6 days.
